# Molecular Control and Application of Male Fertility for Two-Line Hybrid Rice Breeding

**DOI:** 10.3390/ijms21217868

**Published:** 2020-10-23

**Authors:** Muhammad Furqan Ashraf, Guoqing Peng, Zhenlan Liu, Ali Noman, Saad Alamri, Mohamed Hashem, Sameer H. Qari, Omar Mahmoud al Zoubi

**Affiliations:** 1 State Key Laboratory for Conservation and Utilization of Subtropical Agro-Bioresources, College of Life Sciences, South China Agricultural University, Guangzhou 510642, China; pengguoqing@stu.scau.edu.cn (G.P.); zhenlan_liu@scau.edu.cn (Z.L.); 2 Department of Botany, Government College University, Faisalabad 38040, Pakistan; alinoman@gcuf.edu.pk; 3 Department of Biology, College of Science, King Khalid University, Abha 61413, Saudi Arabia; saralomari@kku.edu.sa (S.A.); drmhashem69@yahoo.com (M.H.); 4 Research Center for Advanced Materials Science, King Khalid University, P.O. Box 9004, Abha 61413, Saudi Arabia; 5 Botany and Microbiology Department, Faculty of Science, Assiut University, Assiut 71516, Egypt; 6 Biology Department, Al-jumum University College, Umm Al Qura University, Makkah 21955, Saudi Arabia; shqari@uqu.edu.sa; 7 Department of Biology, Faculty of Science in Yanbu, Taibah University, Yanbu 46423, Saudi Arabia; omaralzoubi73@yahoo.com

**Keywords:** three-line, two-line, single-line, hybrid rice, male fertility or sterility, EGMS, PGMS, TGMS, PTGMS, HGMS, CRISPR/Cas9, apomixes

## Abstract

The significance of the climate change may involve enhancement of plant growth as well as utilization of the environmental alterations in male fertility (MF) regulation via male sterility (MS) systems. We described that MS systems provide a fundamental platform for improvement in agriculture production and have been explicated for creating bulk germplasm of the two-line hybrids (EGMS) in rice as compared to the three-line, to gain production sustainability and exploit its immense potential. Environmental alterations such as photoperiod and/or temperature and humidity regulate MS in EGMS lines via genetic and epigenetic changes, regulation of the noncoding RNAs, and RNA-metabolism including the transcriptional factors (TFs) implication. Herein, this article enlightens a deep understanding of the molecular control of MF in EGMS lines and exploring the regulatory driving forces that function efficiently during plant adaption under a changing environment. We highlighted a possible solution in obtaining more stable hybrids through apomixis (single-line system) for seed production.

## 1. Introduction

Generally, it is a global prediction that the human population will cross 9 billion up to the next decade (www.fao.org). Sustaining food or agriculture production for the rising population is the key challenge and main concern worldwide. Consequently, there is an urgent need to boost food production in an eco-friendly, sustainable, and safe way. Abundant plant species exist around the world, many hundred edible plants are cultivated, out of them only limited species are the main source of food. Rice is also an edible plant species and the staple food cereals for nearly half of the population worldwide, especially in countries that exist in Southeast Asia, East Asia, as well as African regions and is an important driving force to attain sustainable food security (FS) [1]. According to one estimate, only rice production demand will enhance to the 736–852 million tons during 2020–2035. If we examine previous records, the annual rice production increased just 1% while the efforts were carried out during the past two decades [2]. Rice cultivation is feasible in several countries. However, environmental alterations and biotic stress have been the off-putting reason for reaching the targeted high yield. E.g., temperature fluctuations, drought, salinity, soil fertility, pests, microbes, etc., adversely affect rice fertility [3]. Anther development is highly sensitive to the environmental changes during rice flowering, which accordingly poses a serious threat to agriculture by affecting current as well as long-term crop production [3,4,5]. Therefore, crop adaptation requires various changes at the genomic levels and scientists are working consistently to elevate the crop production in major crops and to feed the ever-increasing population [6]. Currently, the availability of the scarce resources and including several environmental constraints, e.g., the emergence of the evolving pests, disease-causing pathogens, and continuously changing environment for farming, is a constant threat to rice cultivation and remain the massive challenge.

### 1.1. Development of Hybrid Rice Technologies

**Figure 1:**
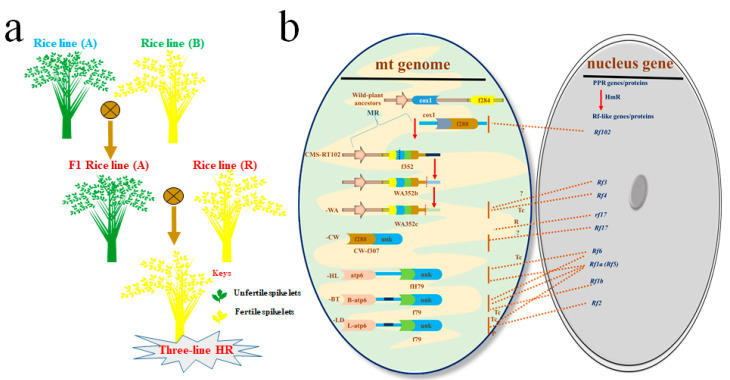
Three-line hybrid-rice technology. (**a**) Three-line HR technology works through three different rice lines, as rice-line A (cytoplasmic-male-sterile line), rice-line B (maintainer line), and rice-line R (restorer line). (**b**) The regulatory factors that can restore rice fertility. Several sequences of the mitochondrial (mt) genome undergo multirecombination (MR) through evolution in rice to generate structural mutations. The flow of sub-stoichiometric due to the variations in copy number of a gene and leading to the emergence of a functional cytoplasmic-male-sterile (CMS) gene. Expanding clusters of the pentatrico-peptide repeat resulted in functional-Rf alleles used for cytoplasmic-male-sterility restoration. The nucleus genes as Rf converse function of CMS gene(s) at transcriptional (Tc) and/or protein (P) levels, but the recessive allele like rf17 is retrogradely (R) upregulated through CMS gene(s). In the figure, MR, HmR, PPR, f284, f288, f352, fH7, f79, -WA, -CW, -HL, -BT, -LD, and unk represent multi-recombination, homologous-recombination, pentatrico-peptide repeat, orf284, orf288, orf352, orfH7, orf79, CMS-WA, CMS-CW, CMS-HL, CMS-BT, CMS-LD, and unknown, respectively.

Previous reports highlighted that crop production was not substantial to support increasing population around the world, a lot of regions of the world became the victim of uprising hunger, in the 1950s. Progressively, rice breeding technology has advanced via the introduction of semi-dwarf varieties (HYV) that were high yielding [7]. The maize and wheat enhancement programs paved the way toward high yield and improvement against lodging and disease resistance through genetic manipulation of the *semi-dwarf* (*sd-1*) gene among various species. These findings enabled the scientists of the International Rice Research Institute (IRRI) to develop the first semi-dwarf rice that has unique properties such as medium height, lodging resistant, a greater number of panicles and grains leading to high yield. During 1966, there was a more dynamic shift that was attained through the green-revolution by genetic manipulation of the rice IR8 variety. It was harboring the *semi-dwarf* (*sd-1*) gene and is known as the miracle rice, termed as international rice 8 (IR8), and enhanced rice yield [8,9]. This discovery and successful manipulation of the sd1 (the semi-dwarf mutant) gene in crops was the first “green-revolution” that facilitated in hunger eradication in the developing countries [10]. To feed the fast-growing population, crop yield was enhanced effectively in several parts of the world by introducing high yield new cultivars during the past few decades. However, rice production with marvelous effects has been in progress since as early as 1926 by investigating heterosis in rice [11,12]. Though the possibility to adopt hybrid-rice (HR) technology was started the first time in 1966 by Yuan Long-ping, later he was pronounced as the father of HR in China [13]. Being a scientist, global FS is an enormous task for human beings. HR gained popularity due to high yield and great advantages as compared to the inbred cultivar/lines [14]. It has also been evidenced that heterosis exploitation is a common phenomenon in crops and the most effective breeding tool against food scarcity worldwide. HR seed production comprises the crossing among two well defined genetically important inbred parental cultivar/lines (one female line and another male line). HR technology provides a better result to improve yield by producing superior quality containing F1 HR over its pure inbred or dwarf lines [15,16]. It is a practical way to enhance rice production by using F1 hybrids, which provided 20–25% more yield benefit over pure rice breeds [13,17,18]. Over time, the tremendous progress in the form of hybrid-breeding technology greatly benefited agriculture by HR with high yield and better tolerance against stressors (e.g., biotic stresses as the diseases, pests, and pathogen infestation and abiotic stresses as the drought, heat, salt, etc.) as compared to the inbred lines/varieties [4,19,20,21]. Rice is a self-pollinated cereal crop; its male fertility (MF, described as the release of the workable gametes or functional pollens that can fertilize female gametes) control demands the male sterility system to generate HR lines/varieties. Male sterility (MS, defined as the production of nonworkable gametes or nonfunctional pollens that can fecundate female gametes), acts as the central player in MF regulation for hybrid-seed production and provides the incredible germplasm to explore rice reproductive development and harness the influence of hybrid-vigor to gain more seed production, as the key breeding tools [22,23]. Overall, MS is grouped into two types such as cytoplasmic-male-sterile (CMS) and environment-sensitive-genic-male-sterility (EGMS) [22]. After the discovery of the male sterility system and its application in HR technology, that originated in China by using male sterility inducing nuclear and cytoplasmic genes to generate cytoplasmic-male-sterile (CMS) lines and CMS also termed as the three-line HR technology, displayed the innovative step towards HR production (Figure 1a) [22,24,25]. 

#### 1.1.1. Three-Line HR Technology

Three-line HR technology works through the three-line-male-sterility system and it requires CMS rice-line (A), the maintainer rice-line (B), and restorer rice-line (R). The rice-line (A) consists of mitochondrial (mt) CMS-influencing gene(s) which lack a workable male fertility nuclear-restorer (Rf) gene. The maintainer rice-line (B) is utilized to maintain sterility trait of rice-line (A) during crossing of (B x A) rice lines. The restorer rice-line (R) is composed of the dominant restorer (Rf) gene and used to produce commercial three-line HR seeds by crossing (B x A) rice lines (Figure 1a) [15,26,27]. The restorer lines contain nuclear Rf genes, which encrypt mitochondria localized proteins such as the pentatrico-peptide repeat (PPR) that suppress CMS defects by acting as the retrograde regulators. The adverse effects on the tapetal and pollen due to CMS proteins, may be liberated via interplay among Rf and CMS genes through innumerable regulatory mechanisms [28]. More specifically, the *rf1a* and *rf1b* in CMS-BT enhance the transcripts degradation of atp6-orf79 and reduce the transcript levels of WA352c, respectively [27]. Whereas, the *rf4* in CMS-WA also decrease WA352c transcripts. The *rf5* (*rf1a*) may play an important role in splicing of atp6-orfH79 in CMS-HL as well as *rf6* by interacting with GRP162 (glycine-rich protein) and OsHXK6 (hexokinase). The *rf2* accelerates transcript degradation of orf79 in CMS-BT and CMS-HL rice lines. In CMS-WA, *Rf3* impedes protein accumulation, while it does not display alterations in WA352c transcripts [29]. Intriguingly, *rf5* and *rf6* have a potential to restore fertility of other CMS rice lines, both originating from the same genetic background (CMS-HL Indica) and this fact suggested a conserved regulatory mechanism of male fertility restoration among various rice types before domestication. Previously, it was reported that hybrid plants composed of *rf5* and *rf6* displayed more stability in male fertility restoration in CMS-HL as compared to plants consisting of a single *Rf* gene [30,31]. Later, it also revealed that *rf6* restoring plants under heat-stress environment exhibited high stability in CMS restoration character than *rf5* restoring hybrid plants. Accumulatively, these findings state that the male fertility restoring mechanism between the *rf5* and *rf6* genes is different as proposed in Figure 1b, the complex mechanisms of interactions among nucleus and mitochondrion have been developed for male fertility restoration in plants. The suggested regulatory factors and origin of identification are described and displayed in Table 1 and Figure 1b, respectively [28]. Although, the greatest merit of this system is the stable sterile rice lines and demerit is the limited availability of the restorer rice line, which make the application of this system limited, and add to the difficulties in the breeding selection in defining a good combination with less probability for seed production of hybrids.

The pure inbred lines are thought to be the essence of the bottom-line of HR technology, due to the parental genetic potential for obtaining the outstanding outcomes of the progenies. Therefore, it is a very difficult and big challenge for rice breeders to choose, cross, generate, and improve the parental line of interest with superior qualities to produce hybrid seeds. This task cannot be dependent only on performance, recruitment of the one progeny harboring gene of interest, and adding up the above-mentioned limitations, insisted search for new breeding technology for seed production that can be simple and more effective as compared to the three-line-male-sterility system. Ensuring food security for a huge population requiring less land and resources necessitates a substantial and persistent increase in agriculture productivity. Here, we are shedding light on the prehistoric continuous progress in improving rice yield. However, the decrease in HR yield has been examined due to abiotic and/or biotic factors like increasing temperature, rice blast, etc. [20,37,38,39]. To tackle this problem, green-super HR-technology has been developed that boosted 20% more grain yield per hectare as compared to HR lines in China [8,38,40].

#### 1.1.2. Two-Line HR Technology

**Figure 2:**
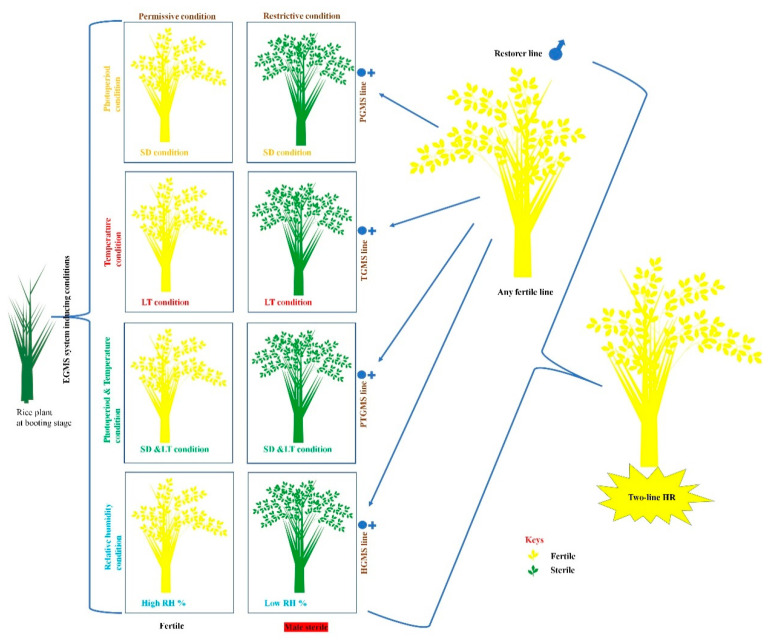
Two-line hybrid-rice technology.

So far, the second innovation was the discovery of the two-line genic-male-sterility-systems to produce hybrid seeds via the two-line HR technology (Figure 2). The EGMS systems regulate male fertility/sterility in EGMS-lines comprising the mutated-gene (mt) via environmental fluctuations. Self-fertilization or environmental factor could maintain the fertility of the EGMS-lines to produce hybrid seeds.

Two-line HR technology is regulated by environmental alterations such as photoperiod and/or temperature and humidity conditions and it also termed as an environmental sensitive-genic-male-sterility (EGMS) system. Therefore, we are reporting that the EGMS system can be categorized as the photo-sensitive-genic-male-sterility (PGMS) system, temperature-sensitive-genic-male-sterility (TGMS) system, and humidity-sensitive-genic-male-sterility (HGMS) system [41,42]. The two-line HR technology gained popularity due to simplicity in creating sterile-rice lines by PGMS, TGMS, and HGMS systems and fertility can be restored via crossing any fertile rice-line and there is no need for a specific restorer-rice line as compared to three-line HR technology. Two-line HR technology is a key breakthrough in crop-breeding history and originated in China. Research evidence revealed that two-line HR has several merits. The first salient feature does not require specific maintainer lines. The sterile line plays a dual role as the maintainer and sterile-lines during the crossing and this trait makes it less cumbersome and facilitates breeding technology by simplifying, shortening breeding cycle, and reducing the cost of labor. Secondly, it has a broad range of restorer lines and does not need specific restorer lines/genes. Due to its versatility, almost all rice varieties can be utilized as restorer lines, enabling the crossing quite freely, which is conducive to utilizing the heterosis among subspecies and boosts the yield of the HR combinations. Its merits also encompass HR grain quality and resistance against stressors [24,37,43]. These merits significantly favor the acceptability and multiplication of the two-line HR genetic manipulation across the globe. Furthermore, it has great potential for improvement in yield, quality, and resistance against stressors in HR [20,44]. Recent statistics released by the National Rice Data Centre showed there were cultivated 73 and 40 varieties/types of the three-line HR and two-line HR, respectively, during 2009 to 2019 and only two-line HR accounts for 53% [45]. The demerits of the two-line HR require the specific environmental conditions to regulate fertility of the sterile line for fertility transition, yet environmental conditions may fluctuate commonly and cannot be controlled by human activities, it may result in the abnormal or failure of the seed production. These fluctuations may influence the photo-sensitive-sterile line and/or thermo-sensitive-sterile line and humidity-sensitive-sterile line. First, the critical-sterility-inducing-temperature (CSIT) describes the temperature at which the thermo-sensitive-sterile line deviates from male fertility (MF) to complete male sterile (MS) and is the regulatory factor for a thermo-sensitive-sterile line. Many rice varieties display various sterility regulation initiation temperature in the genetic background, and several varieties particularly the Japonica rice background have more diversity in sterility regulation temperature [42,46]. If temperature is less than the regulation initiation temperature of the sterile line (thermo-sensitive-sterile line) during seasonal seed production, it will result in selfing and seed setting that can produce impure or even lead to the failure of seed production. Previously, it has been cited that sometimes seed production of the two-line HR fails due to alterations in the ambient temperature. Consequently, such facts cause direct economic losses, and have exceeded 100 million yuan on multiple occasions, yet indirect losses have not been estimated [47]. Therefore, it is an increasing threat and big challenge in two-rice HR technology to create the temperature-sensitive-sterile line to attain the stable dual feature containing the MS responsive gene with a low initial temperature for fertility regulation. Secondly, the photo-sensitive-sterile line is reported theoretically more stable than the temperature-sensitive-sterile line because the photoperiod is considered more stable than temperature influencing environmental conditions. However, the regulatory mechanism of photosensitivity is more complex, and somehow may be influenced by light as well as the temperature at the same time or may be controlled by many genes at the same time [42,48,49,50,51]. The photo-sensitive-sterile line is susceptible to drift in the temperature of the initiating point of the sterility in the offspring, so the genetic stability is unstable, and leads to the insecurity in seed production of the photo-sensitive-sterile lines [52,53]. Finally, a humidity-sensitive-sterile line regulated by humidity is also a hard task to control under natural conditions, and its cultivation area is limited to the arid areas for seed production [54,55]. Tapping into the advantages of HR relies on comprehensive studies to secure seed production and sustain crop yield.

## 2. Environment-Sensitive Genic Male Sterility Systems

The fertility transition in two-line HR occurs due to regulation of the EGMS (PGMS, TGMS, and HGMS) systems via environmental alterations (Figure 2) [22,24,25,45,56]. EGMS genes along with MYB TFs, noncoding RNA i.e., RNase ZS1, E3 ubiquitin ligase, UDP glucose pyrophosphorylase, and leucine-rich-repeat receptor-like kinase, have been described in Arabidopsis and rice [43,46,49,57,58,59,60,61]. Still, there is not enough evidence regarding how temperature fluctuations regulate fertility in EGMS lines due to mutations among genes. The plant scientists have become inquisitive in exploiting detailed mechanisms since the creation of the EGMS lines [25]. The main concept of EGMS relies on the creation and understanding of the mutation in EGMS-related genes that make the development of male gametes more responsive to environmental fluctuations. The creation of the EGMS-lines via EGMS systems in two-line HR has great importance in response to environmental fluctuations. Therefore, we are elaborating on each EGMS system responsive genetic and environmental factor that can influence seed production in two-line HR (EGMS-rice lines). This research field has been attracting scientists globally and remains to be illustrated in broader perspectives. Herein, we describe in detail the systems and possible use for creating EGMS-lines in two-line HR.

### 2.1. Photoperiod-Sensitive-Genic-Male-Sterility (PGMS) System

Photoperiod regulates several processes of plant growth and development. It is also well known that day-length (DL) variations (photoperiod) influence greatly flowering events in plants. Several higher plants switch on the reproductive phase from the vegetative growth phase by utilizing the DL as the environmental cue and interrupted photoperiod causes defects in floral transition among plants [62]. It also has been described in many plants that fertility transition due to the male sterility development is affected by photoperiod, especially pollen development needs a critical DL. Yet, not clear understanding exists to investigate the development of the male reproductive part in many plants. Photoperiod-sensitive-genic-male-sterility (PGMS) system defines the ability of the male gametes to be male sterile when DL is greater than the critical limit and restore male fertility under less DL than the critical limit of DL. The photoperiod (DL) is considered the key regulator of the PGMS-system. PGMS-lines created via the PGMS-system are male sterile under long-day (LD) conditions and restore male fertility under short-day (SD) conditions [58]. The detection of natural PGMS mutant in 1973 from *Oryza sativa* (spp. Japonica) cultivar Nongken58 (NK58) that was designated as Nongken58S (NK58S), laid the initial foundation for developing or examining rice PGMS-lines [51,63]. The described prominent character of NK58S is complete retention of male sterility or partial/complete fertility, when day-length is longer and shorter than 13.75 h at the stage of anther development, respectively [58,64]. NK58S was extensively used in crossing or transferring PGMS traits into Japonica and elite Indica rice-lines at various rice research centers in China [56]. Nowadays, this thought exists that PGMS in NK58S is controlled by *pms1*, *pms 2*, and *pms 3* (Table 1 and Table 2) [51,62,65,66,67,68,69]. There is also evidence that PGMS lines are affected due to temperature, not only by photoperiod. E.g., the Peiai64S (PA64S) line was generated by crossing the NK58S line and Peiai64 line as the female line and male line, respectively. Initially, it was believed that the fertility of the PA64S, regulated through the PGMS system and more than 10 varieties of the two-line HR, were generated in China since 1996. The HR generated by PA64S (female), was the most popular practice in two-line HR technology, and Liangyoupei was declared the annual champion in HR production [45]. Later, it was observed that fertility of the PA64S is also influenced by temperature, not only by day-length. These findings lead the scientists towards the discovery of the temperature-sensitive-genic-male-sterility (TGMS) system. Hence, it was suggested by investigating the fertility transition of the PA64S line, that showed the dual trait of the EGMS as TGMS and PGMS, it displays sterility when high-temperature (HT) was >23.5 °C and long-day (LD) ≥14 h conditions and restored fertility at low-temperature (LT) ≈ 21–23.5 °C and short-day (SD) <14 h conditions during the stage of anther development [40,42,49]. Further, it was also discovered that inherent defects of the day-length/temperature-sensitive-sterile line of the NK58 with the genetic background of Nongken, harbors temperature drift phenomenon in defining the initiating point of the male sterility. This defect results in huge economic losses due to the failure of seed production and raised critical aspects and hindered further hybrid breeding development in this case [70,71,72]. Then, the search for new, more stable germplasm for generating PGMS lines was triggered in two-line HR. In contrast to the conditions required for creating PGMS-lines, PGMS-lines’ fertility can be restored via changing DL, e.g., D52S and YiD1S demonstrate male sterility under SD (<12.5 h) conditions and restore fertility under LD (>13.5 h) conditions, it is designated as reverse PGMS (rPGMS) lines (e.g., *rpms1* and *rpms2* genes utilized) [73,74,75]. However, Zhang et al. [75] reported csa mutant, rPGMS lines 9522^csa^ and JY5B^csa^, as the rPGMS lines that can be the stable germplasm for generating PGMS lines in two-line HR [76]. PGMS system can be used in temperate regions for PGMS-lines production where marked differences in day-length exist during rice growing seasons [15,76]. The PGMS system might be the right option for PGMS-lines seed production in subtropical and tropical regions like Sanya, Hainan, and Shanghai (it has LD condition in summer) provinces of China [75].

### 2.2. Temperature-Sensitive-Genic-Male-Sterility (TGMS) System

**Figure 3:**
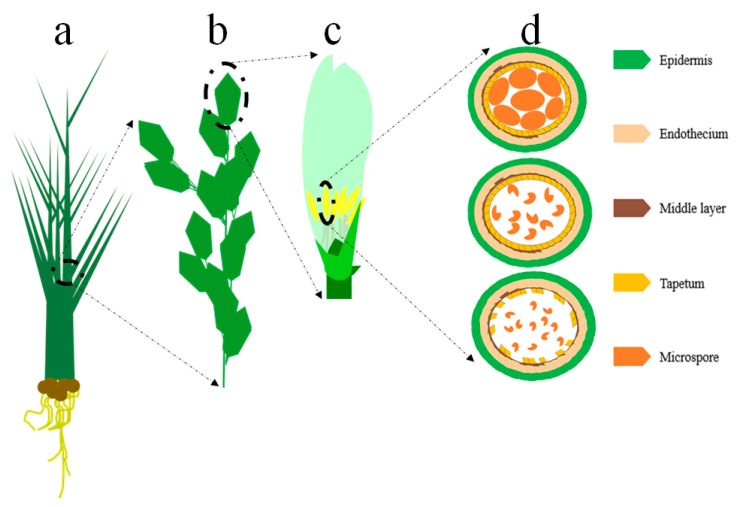
Examination of the male reproductive part in rice plant. (**a**) Rice plant at the booting stage. (**b**) Rice flower. (**c**) Dissection of the single-spikelet reproductive parts. (**d**) Proposed ultra-structure examination of the anther development. Upper-circular-part displayed normal anther development in rice, middle-circular-part showed abnormal anther development due to disruption of the microspores, and lower-circular-part showed abnormal development of tapetum and microspores during anther development. These abnormalities in anther development lead to the male sterility phenotype in rice.

Thermo-sensitive-genic-male-sterility (TGMS) system refers to the ability of the male gametes to be sterile or fertile at a higher or lower temperature than the critical point. The TGMS system induces male sterility to male fertility through temperature variations at the critical anther developmental stage of the crop [80,93]. For the first time in 1986, the TGMS-lines were discovered in China during the extensive study of the cytoplasmic-male-sterility (CMS) restorer line-5460. The 5460S was planted under low and high temperature, regardless of day-length (photoperiod) in the growth chamber, it revealed normal fertility at low temperature and sterility variations at high temperature. It was the first successful application of the TGMS system to develop TGMS-lines in three-line HR [94]. Afterward, in two-line HR, the TGMS-line, Annong S-1 (AnS-1) originated from F3 population (cross chao-40/H285/6206_3) as a result of the spontaneous mutant in 1987 [46,80]. Further studies demonstrated that pollen-mother-cell (PMC) formation, as well as meiosis stages, are induction detection sites for TGMS because at high-temperature wrinkled or abortive pollen grains were produced due to abnormal meiosis in microspore-mother-cells (MMC) (Figure 3) [46]. Besides, other TGMS-lines were also reported from Japan, The Philippines, India, and Vietnam [95,96,97,98].

Mostly, reported TGMS-lines or mutants induce male sterility at high temperatures and male fertility at low temperatures [99,100,101]. The stated TGMS genes/lines are *tms1*, *tms2*, *tms3*, *tms4*, *tms5*, *tms6*, *tms7(t)*, *tms8*, *tms9*, *tms9-1,* and *tms10* [49,81,82,84,86,89,92,102,103] and Zao25S, Lu18S, N28S, 95,850ms, XianS, Zhu1S, Meixiang851S, and HD9802S [104,105,106,107,108,109], that provide useful material for two-line HR production (Table 2 and Table 3). Intriguingly, the reverse phenomena were also observed such as male sterility induced at low temperature and fertility restored at high temperature. Such kinds of TGMS rice-lines are termed as reverse TGMS (rTGMS) lines. Herein, the reported rTGMS genes/lines are *rtms1*, Diaxin-1A, and IVA and the mutant of Indica-rice variety 26-Zhaizao from China and JP-38S from India [91,110,111,112,113]. The *tms5* is an important factor that regulates thermosensitive sterility among many TGMS lines. Although, it deflects variations in sterility inducing temperature between different genetic background germplasm. Initially, two sterile lines were generated by using the *tms5* temperature-sensitive sterile gene. Afterwards, it was found that the sterility inducing temperature, is quite stable among the offspring, and this outcome helps the scientists to replace, step by step, the genetically unstable sterile lines derived from NK58S. Before, the sterile gene derived from Nongken 58S was in use, but now the putative *tms5* gene is 5 further study in two-line HR technology. Recently, two more genes (TMS10 and TMS10L), encoding leucine-rich-repeat receptor-like kinases have revealed redundant function in controlling rice tapetal and pollen development. The tms10 mutants displayed a TGMS trait, by showing male sterility at high temperature and resorted fertility at low temperature [43]. The TGMS system is highly temperature-sensitive, any fluctuation in the temperature range (22–24 °C) could cause a severe effect on seed setting via sterility [42,114]. The TGMS-system may be recommended in those countries that have tropical and subtropical rice cultivation areas, where persistent distinctive temperature fluctuations exist across the land as well as seasons. Particularly, near the equator among smaller tropical regions, where low temperatures exist in hilly areas [15,115]. The changing climate has been adversely affecting global agriculture production [116,117,118]. TGMS lines are the most vulnerable rice germplasm due to spontaneous environmental temperature rise or fall that causes a severe dramatic decline in seed production of HR [65,70,71]. Therefore, it is an urgent demand to generate more stable temperature-sensitive sterile lines through finding and cloning, and analyzing the critical sterility inducing temperature (CSIT) as well as molecular mechanisms to address the current problem of rising CSIT, and to achieve sustainable seed production and ensure safety of the temperature-sensitive sterile lines.

### 2.3. Simultaneously Photoperiod and Temperature Influence PGMS and TGMS Systems in PTGMS Lines

The fertility to sterility transition is regulated due to the influence of the PGMS and TGMS systems in HR through a strong combination of photoperiod and temperature in a certain duration/day-length and limit at a specific developmental stage during the reproductive phase, i.e., the male gametes to be male sterile at high-temperature (HT) and short-day-length (SD) combination compared to a critical limit and reverted to fertility at low-temperature (LT) and long-day-length (LD) combination compared to the critical limit. Comprehensive study of NK58S, a PGMS mutant showed that phenotype of male sterility or fertility occurred due to significant interplay among temperature and photoperiod, not only the result of the alteration of photoperiod or temperature [49,121]. Concrete findings of the scientists suggested applicable evidence of the strong combination among temperature and photoperiod to induce sterility/fertility in PTGMS-lines [24,49,50]. Zhou et al. [49] suggested that Pei’ai 64S showed male sterility under LD and HT conditions and termed it as reverse PTGMS-line. In China, it is believed that more than 95% of the EGMS-lines employed in HR creation were resulting from three separate genetic resources, i.e., PGMS-lines from NK58S and TGMS-lines from AnS-1 and Zhu1S. Several EGMS-lines originating via NK58S were PTGMS-lines/even TGMS (as Guangzhou 63S), the cause of the underpinning mechanism for such unpredictable variations has not been well explained [122,123]. Nowadays it is believed that some EGMS lines are sensitive to photoperiod for the appearance of male sterility/fertility in rice. NK58S gene was used to develop Indica background rice-lines in China that could be classified as PTGMS lines (Table 3) [50,124,125]. Mengchen et al. [126] genetically characterized 208 rice lines with the PTGMS trait and that belong to the Indica background [126]. The food increasing demand and rice cultivation practices enhanced use of the HR varieties. The registered two-line HR combinations of P/TGMS had been 427 lines in China and covered ≈ 20% of the total cultivation area of the HR [45].

### 2.4. Humidity-Sensitive-Genic-Male-Sterility (HGMS) System

Strict conditional limitations for developing new HR lines via PGMS and TGMS systems, open a new door to introduce another system of EGMS; it is called the Humidity-sensitive-Genic-Male-Sterility (HGMS) system [41,54]. HGMS means that the development of male gametes is susceptible to humidity, which displays male abortion under low humidity conditions and male fertility under high humidity conditions. In the HGMS system, relative humidity (RH) is the key driving force that influences rice fertility/sterility regulation [54]. Meanwhile, it is the only reported system which is a temperature and/or photoperiod independent operating system to create two-line HR. The HGMS system might be a potential way to generate HGMS germplasm in zones somewhere with RH > 60% [54,120]. It is a prominent character of it, to produce HR seed (F1 hybrid) under prolong high-humidity (HH) condition regions via selfing and transition from fertility to sterility under low-humidity (LH) condition areas. So far, very little literature is available about the HGMS system and the stated HGMS lines/mutants are 01v635, 02v750, 02v762, 03v645, and hms1 [41,54,120]. Such HR germplasm could be a potential source to exploit underlying mechanisms. Chen et al. [41] reported that hms1 mutant is the HGMS-line and speculated allelic mutation, and it might also be a good reference for improving resistance in crops against stresses such as drought, high-temperatures, etc. The merit of the HGMS system indicates that it can be regulated by proper irrigation at a critical stage of the crop to secure HR seed production [127]. Therefore, HGMS system/lines (Table 3) could potentially be used in HR breeding zones where the RH is more than 60%. While, unexpected alteration in humidity caused by rain, especially at the critical stage (flowering), can create problems with HGMS-based seed production of hybrids. Therefore, HGMS lines can be the suitable choice for arid regions, such as in Urumqi, Xinjiang, China, where RH above 80% was observed between 2005 and 2014 [54,55].

## 3. Importance and Application of Two-Line HR for Seed Production of EGMS-Lines

Two-line HR technology is the discovery and effective utilization of the EGMS systems and/or lines, and is getting more attention in agriculture globally [46,49,93,128,129,130]. With the fast pace, this field of research is creating more and more applicable germplasm resources all over the world [126] that could be used to explore underpinning mechanisms in EGMS-lines and has remained poorly understood. Comparative significance of the two-line system over the three-line system, shows great advantages e.g., EGMS-lines (Table 3), can produce seed itself under permissive conditions and also have the potential to generate HR under restrictive conditions [24,28], this character reduces the cost of labor, time, and production resources. EGMS-lines’ fertility can be restored by using any fertile rice variety for F1 hybrid seed production. During the last three decades, it gained tremendous significance because it is simple and has broad-spectrum application in rice breeding for exploiting heterosis in hybrid-seed production. The two-line HR lines or combinations in China were cultivated at a larger scale. After this, several HR lines/varieties were developed and released by different institutions and a persistent increasing trend was observed in the planting area of two-line HR in China [131].

### 3.1. Molecular Regulation of EGMS Lines

Two-line HR is gaining high praise due to 5–10% high yield and easiness in seed production as compared to three-line HR and the discovery of EGMS systems has also flourished and made it an ideal replacement to the CMS [132,133,134]. Unrevealing molecular mechanisms and determining underlying factors could be a way to introduce additional EGMS system/s for more genetic resources in a variety of crops that will provide a basic foundation for new hybrids.

#### 3.1.1. EGMS Lines Are Influenced by Genetic and Epigenetic Alterations

**Figure 4:**
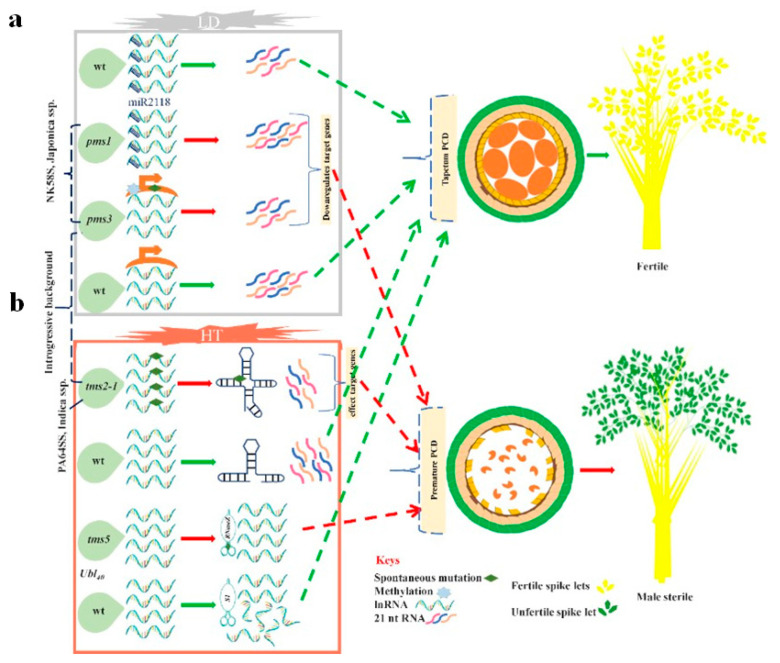
Regulation of the male fertility of EGMS-lines in two-line HR through photoperiod and temperature alterations. (**a**) The NK58s (Japonica genetic background) comprise the single nucleotide polymorphism (SNP) within two long-noncoding RNAs (lncRNAs), pms1 and pms3. The SNP in Pms1 induces miR2118 binding and enhances the small RNA (21nt) processing under long-day (LD) conditions that downregulate the target genes responsible for tapetum programmed cell death (PCD). Additionally, the SNP in the promoter region of the pms3 that elevates the DNA-methylation reduced pms3 transcription under LD conditions proceeding the premature-PCD in the anther (PGMS). The SNP within pms3 in tms12-1 regulates TGMS in PA64s (the NK58s generated line with Indica genetic background). (**b**) The mutation in the TMS5 gene generated a tms5 line (TGMS) which encrypts RNase Z (RNase Z^S1^) short form. RNase Z^S1^ produces mRNAs splicing that encodes Ub_L40_ protein. The mRNAs of the Ub_L40_ under high temperature (HT) could not be spliced and elevated accumulation levels of mRNAs, resulting in male sterility in the tms5.

Genetic background plays a very important role in understanding mechanisms that can be responsible for creating new HR. Previous literature showed that a single/two genes or even more genes could cause genetic-male-sterility based on genetic resources as well as the environments. For example, in the case study of the crossing of the Japonica and Indica with Nongken 58S, as a result, all siblings of the F1 were fertile [16]. When reciprocal crosses of F2 to Nongken 58S were carried out, the outcome suggested that a single recessive gene implies male sterility [135]. Further support was obtained via investigating a cross between Nongken 58 (fertile) and Nongken 58S (sterile) and similar results found in the F2 population under LD conditions due to a single recessive gene [66]. Even single-locus segregation of EGMS-lines has also been described in various lines as the cause of TGMS lines that were grown under LD and HT field conditions [46,83,119,136]. In some studies, two recessive-genes segregation was discovered after crossing Nongken-58S to an Indica (variety). The F2 segregation population revealed a pollen fertility ratio of 15 fertile to 1 sterile [65]. Many Indica background male sterile lines indicated parallel segregation genetic ratios like Peiai64S [78,87,88]. Some genetic-male-sterile populations demonstrated that segregation followed a continuous distribution or bimodal type [137,138]. Previous studies showed that a single-recessive-gene control TGMS-lines’ traits and induce male sterility via temperature variations like *tms1*, *tms2*, *tms3*, *tsm4*, *tms5*, *tms6*, *tms6(t)*, *tms8*, and *tms9* genes positioned on the chromosomes (chr) chr 8, chr 7, chr 6, chr 9, chr 2, chr 5, chr 10, chr 11, and chr 11, respectively. Reported PGMS-lines’ traits controlled by *pms1* on chr 7, *pms2* on chr 3, and *pms3* on chr 12 that change fertility to sterility phase due to photoperiod [65,66]. PTGMS-line trait is regulated through temperature and photoperiod. The genetic control of sterility in PTGMS-lines is complex because of regulation through major and/or minor effects of multiple genes during fertility to the sterility phase transition, that exist on chr 3, chr 5, chr 6, chr 7, chr 11, and chr 12 [16]. In two-line HR as PA64S (PGMS-line) revealed that levels of DNA-methylation were high under LD and HT conditions (Figure 4a,b) suggesting that fertility to sterility transition was regulated epigenetically in EGMS-lines of rice [139]. A recessive mutation of OsOSC12/OsPTS1 that was positioned at chr 8 induces sterility in E157, S1708, and S4928 mutants at low RH (<60%) and fertility at high RH (>80%) in a HGMS-line [54]. Chen et al. [41] reported that the *humidity-sensitive-genic-male-sterile 1* (*hms1*) gene that presents on Chr 3 revealed potential genetic material for the production of HGMS-lines in two-line HR [41]. Despite the decades of studies, still gaps exist, e.g., the research conducted in the US by using male sterile line 2008S (Indica background originated from China) showed two/three recessive-genes implied male sterility relying on cultivation year and site. The F2 population cultivation derived from a cross (2008SxCL131) showed a three-gene model under Stuttgart-Arkansas USA when the same population grown in Crowley-Louisiana USA revealed the two-gene model in 2013 [128] and such findings need more investigations to finetune the underpinning mechanism.

#### 3.1.2. Regulation of the EGMS Lines by Noncoding RNAs and RNA Metabolism

##### The tms5 Regulates RNA-Metabolism

Literature reflects that splicing abundance of the precursor-messenger-RNA (pre-mRNA) and dependent translation plays an important role in plant adaptation under stress [140,141]. Experimental data disclose the multifaceted switches of male fertility to sterility transition and vice versa under the fluctuating environment. The role of temperature and/or photoperiod in the regulation of male organ development largely remains to be explicated. The *thermo-sensitive-genic-male-sterile 5* (abbreviated as *tms5* and other names used for it are TMS-X, PTGMS2-1, and TMS9) gene is the only abundantly utilized temperature inducible gene source for the genetic manipulation to create TGMS-lines in HR. The discovery of a single conserved mutation in eukaryotes was identified as ribonuclease Z (RNase Z). It is also referred to as RNase Z^S1^, and believed it works independently in temperature variations, and remains detectable at restrictive and permissive temperature conditions in many tissues. The *tms5* also encrypts the conserved protein RNase Z^S1^ that participates in messenger-RNA (mRNAs) processing, into many fragments of the ubiquitin-fusion-ribosomal protein L40 (Ub_L40_) genes. The *tms5* may also react indirectly against temperature alterations via potentially degrading the Ub_L40_ mRNA, not directly affecting the levels of mRNA or protein [46]. A comprehensive study, for exploring the mechanism of male fertility in EGMS lines against environmental fluctuations in two-line HR, indicated that during anther development, TMS5 mRNA accumulates more in PMC and TMS5 protein confined in the cytoplasm [46]. RNase Z^S1^ can slice mRNA that translates three ubiquitin-fusion-ribosomal-protein L40s (Ub_L40_) that are mainly expressed in PMC and can be induced by temperature. In tms5, a point transition (C-to-A) at location 71nt of TMS5, creates a premature stop codon. Under permissive temperature (LT), Ub_L40_ mRNA levels were low and result in no defects in anther leading to the normal pollen grain production in tms5 lines (Figure 4b). In contrast, under the restrictive temperature (HT), *TMS5* is unable to process mRNAs of Ub_L40_ in tms5 lines at HT, and these elevated levels of mRNAs of Ub_L40_ cause defective pollen growth and result in male sterility [46]. Zhou et al. [49] also revealed a substitution (C-to-G) in the DNA sequence of NK58S, and discovered that this point mutation causes PMGS (Japonica background) and TGMS (Indica background) due to loss-of-function of the sRNA (osa-smR5864m).

##### Transcriptional Regulation of the EGMS Lines via Noncoding RNAs

A cell begins transcriptional regulation when DNA to RNA conversion (transcription) takes place and so orchestrating gene modulation. The gene can operate in various ways, by varying the number of RNA copies that remain transcribed as well as the sequential control once the transcription of the gene occurred. This control mechanism permits the organism to interact against several intracellular and extracellular stimuli and accordingly mount feedback. Such examples consist of the mRNA production that encrypts genes/enzymes to attain adjustment according to the fluctuating environment by fabricating products of a gene during the specific stage of cell development [142]. The microRNAs (miRNAs) comprise the small long-noncoding RNAs (lncRNAs) containing ≈ 22 nucleotides (nt) length, which are involved in gene expression regulation through the degradation of the respective target mRNAs. The lncRNAs are key regulators in transcriptional processes and present in huge amounts in plants. The lncRNAs are responsible for plant growth and adaptation to the fluctuating environment [143]. Little is known about the role of variations at lncRNAs loci for creating morphological and developmental deviations in EGMS lines. Indeed, the PMS3 and P/TGMS12-1 induce traits of PGMS and TGMS within NK58S and PA64S, respectively, and may belong to the same gene that is a long-noncoding-RNA known as long-day-specific-male-fertility-associated-RNA (LDMAR) [49,144]. An adequate quantity of the LDMAR is necessarily required to induce male fertility under a long-day environment. Interestingly, the natural spontaneous mutation (G to C) difference by a single nucleotide polymorphism (SNP) among the lncRNA of the NK58 and NK58S altered the secondary structure of the RNA and induces EGMS trait via a complex regulatory mechanism comprising the transcriptional regulation intermediate through small-RNAs (sRNAs) as well as DNA methylation. The SNP mutation caused heritable enhanced DNA methylation within the promoter of the LDMAR, leading to a reduction in transcript levels under a long-day environment [58,139,144]. These decreased LDMAR transcript levels cause premature PCD during the development of anther under a long-day environment, so triggering the male sterility and demonstrated the PGMS trait in EGMS lines.

#### 3.1.3. Post-Transcriptional Regulation of the EGMS Lines via Alterations in RNA Expression

More recently, miRNA-based regulation of gene expression at the post-transcriptional level has come into the focus of research efforts on flowering-related pathways [145]. In rice, nine TGMS related loci have been mapped. The spontaneous PGMS mutant (NK58S, Japonica background) was fertile and sterile under SD and LD conditions, respectively. The NK58S (Indica background) revealed a TGMS trait with a 24 °C threshold temperature [49,58]. The genetic analysis exposed that NK58S’s trait of PGMS due to two loci as photoperiod-sensitive-genic-male-sterility 1 (*pms1*) and *pms3*. The *pms1* translates lncRNA via the phasiRNAs-producing locus, which produces *pms1t* transcript generated through miR2118 to create 21nt phasiRNAs. The expression level of *pms1t* is enhanced under LD condition throughout the development of pollen-mother-cells (Figure 4a). The SNP in *pms1t* adjacent to the recognition site of miRNA2118 implies alleviation of phasiRNAs that was thought fertile to male sterile transition site [59]. The phase-specific (phasiRNAs) as reproductive stage-specific miRNA2118 (21nt) and miRNA2275 (24nt), may perform vital roles during micro-gametogenesis in maize as well as rice [59,146]. The *pms3* under LD conditions translates 1236nt lncRNA that promotes pollen growth and reduction in transcript levels was found due to elevated siRNA-directed methylation inside *PMS3′*s promoter region because of a single SNP (Figure 4a) [58,144]. Previously, Wu et al. [147] reported that transcriptomic data revealed that there are 24 conserved microRNAs (miRNAs). The miRNAs are expressed differently during pollen development among male sterile lines and may interact with kinases, MYB transcription-factor (TFs) family proteins, and PPP domain comprising proteins, which play an important role in reproductive processes particularly anther development regulation. The miR159 in plants regulates the transcript expression of the GAMYB TFs or GAMYB-like TFs genes, which regulate microsporogenesis and anther development. When a mutant with GAMYB deletion was studied, it displayed sterility due to the premature programmed cell death (PCD) or degradation of the tapetum during anther development. The miR159 was overexpressed in cereal and *Arabidopsis thaliana* exhibited male sterility. Additionally, the miR159 may influence the expression of the miR167 and miR319. The miR319 and miR159 inhibit the transcript expression of the TCP4 and GAMYB TFs, respectively [147]. Wu et al. [147] also reported that the miR172, miR158, miR169, miR4399, and miR9473 were expressed in PA64S under treatments of the high-temperature and low-temperature stresses, respectively. The post-transcriptional regulation was observed by Jiang et al. [148] when the MYOSIN XI B gene in *Oryza sativa* mutated via Ds or insertion genetic manipulation and is termed as OSMYOXI mutant. The fertility of the OSMYOXI mutant is regulated by the photo-sensitive-genetic-male-sterility system. The OSMYOXI mutant was sterile under SD conditions due to abnormal development of pollens but displayed partial normal pollen development in LD condition. The transcript of OSMYOXI was observed in the whole anther under LD and SD conditions. However, the OSMYOXI-Gus-fusion protein was visualized in the anther’s epidermal layer under the SD condition. Consequently, the pollen development of the mutant was disrupted and led to the male sterile phenotype. In contrast, the fluorescent protein signals were detected in whole anther (epidermal, endothecium, middle, as well as tapetum layers) and displayed normal anther development under LD conditions. These outcomes showed that the OSMYOXI mutant is photoperiod responsive and deflects fertility and sterility under SD and LD conditions, respectively. Jiang et al. [148] suggested that OSMYOXI regulates pollen development through post-transcriptional modulation via 3′-UTR as well as sequences of the dilute (DIL) domain of the gene at the cellular level by photoperiod stimuli [148].

#### 3.1.4. The mRNA Splicing Regulates Fertility Transition in EGMS Lines

Although many EGMS genes have been reported and mapped on chromosomes in rice, still several genes have not been cloned for exploring the regulatory mechanism of the EGMS lines in HR. Chen et al. [57] carried out a comprehensive study of Ugp1 rice and demonstrated that mRNA splicing is influenced by temperature alterations and the accumulation of the spliced or nonspliced mRNA may be the molecular cause of the fertility reversion in the TGMS line as Upg1. The Upg1 plays a vital role in PMC meiosis and the development of microspores in rice. When Upg1 was silenced through the RNA-interference (RNAi) or cosuppressed, it caused defects in the development of the pollen wall due to interrupted callose deposition. As a result, PMC degenerated at the meiosis initiation stage and leading to male sterility in HR. The population of the transformants of Ugp1-OX lines segregated among two subpopulation groups. One subpopulation group showed complete suppression of the endogenous expression of Ugp1, indicating its cosuppression, and this population was termed as the cosuppressing plants. Importantly, it contains aberrant intron that was “longer-than-full-length” mRNAs of the Ugp1, these are derived from the Ugp1-OX transcription via primary transcripts’ un-processing that existed within cosuppressing plants. There were no phenotypical variations among cosuppressing plants at the vegetative-growth stage. However, cosuppressing plants deflected complete male sterility at the reproductive-growth stage during the natural season, but these plants may display fertility under the autumn season. These results suggested that fertility transition under SD is controlled via temperature in cosuppressing plants, not by photoperiod and designated as TGMS. Furthermore, Chen et al. [57] suggested the cause of fertility transition due to the accumulation of the proteins (UGPase) within florets in cosuppressing plants under low-temperature. More experimental evidence reflected that mRNA splicing of the Ugp1 was significantly regulated by temperature alterations when florets of cosuppressing plants were cultured at low-temperature, and there was more accumulation of the spliced mRNA of the Ugp1 than florets cultured under high-temperature. This proper spliced mRNA of the Ugp1 permits a high level of the UGPase in florets of cosuppressing plants and thus leads to the fertility phenotype [57]. As a result, the overexpressing rice of Upg1 exhibit the TGMS-line trait under normal temperature due to the nonsplicing of endogenous Ugp1 transcript and efficient splicing of Ugp1 mRNA takes place at LT that display male fertility. These outcomes explored a possible molecular mechanism of the fertility transition in the TGMS line (cosuppressing plants).

#### 3.1.5. Metabolism of the miRNAs and Structural Substances Regulate the EGMS Trait

**Figure 5:**
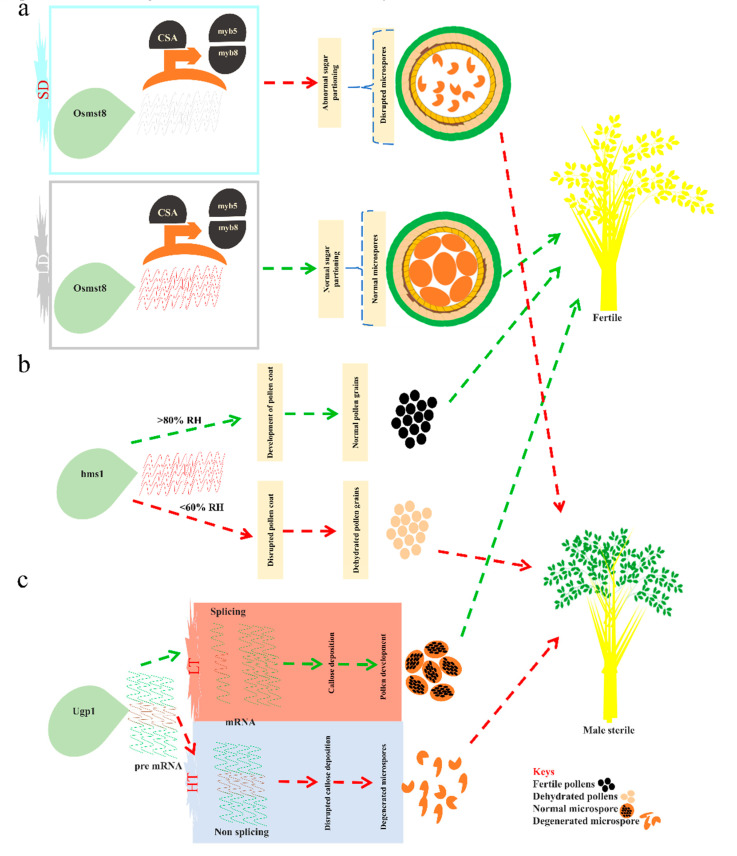
Transitional regulation of male fertility and sterility in EGMS-Lines. (**a**) Regulation of sugar partitioning in anther via the carbon-starved. The transcription factor CSA is a key player for sugar regulation from leaf to anther through direct regulation of transporter OsMST8 for normal anther reproduction. Under a short day, CSA mutation can lead to downregulation of OsMST8 transcription and be unable to transport sugar from flag leaves to anthers, resulting in male sterility. While, long-day (LD) conditions along with other regulators might switch regulation of this process and lead to normal anther development and male fertility. (**b**) The hms1 mutants regulate male reproduction under varying relative humidity (RH) percentage. It showed male sterility at low RH (<60%) and restored male fertility at high RH (>80%) via normal development of the anther. (**c**) Functional regulation of UPD-glucose pyrophosphorylase 1 (Ugp-1) under fluctuating temperature. Under high-temperature (HT) Ugp-1-overexpression rice plants revealed a high accumulation of nonspliced transcript of Ugp1 leading to male sterile plants, whereas under low-temperature (LT) successful splicing of Ugp1 transcript restored male fertility and normal seed setting.

The proper development and regulation of the male gametes in plants permit more stability among fertility to sterility transition that can play a key role in exploring the molecular mechanism of MS systems. The microRNAs (miRNAs) facilitate plant growth and adaptation against environmental alterations [143,149]. Currently, it has been demonstrated that certain biosynthetic and metabolic pathways may be interlinked with the development of anthers [147]. These pathways can involve the secondary metabolites and phenylpropanoid biosynthesis, sucrose, starch as well as the metabolism of the sphingolipids. Wu et al. [147] conducted a comprehensive study to exhibit that variously expressed miRNAs regarding the genes of interest have participated in the metabolic pathways as well as the secondary metabolites’ biosynthesis. The noteworthy pathways are sucrose and starch metabolism. Both sucrose and starch metabolism deliver building material for whole plant growth and development, and particularly the aggregation of the dissolvable sugar directly regulates male fertility to sterility transition. Another pathway is sphingolipid metabolism that is regulated via target genes of the miRNA and sphingolipids also regulate programmed cell death (PCD) and male fertility. The proteomic pathways include proline and arginine metabolism that play an important role in plant fertility such as the conversion of the aspartic acid to proline and retardation of the glutamic acids considered the possible reason for male sterility among rice sterile lines. Further hypermethylation data of the PA64 revealed that LOC_Os09g38100 and LOC_Os06g40200 perform phosphate-carrier protein mitochondrial-precursor and calcium-binding mitochondrial-carrier annotated function, respectively, displayed more methylation levels in the PA64S (S) than the PA64S (F) [147,150]. Chen et al. [57] carried a study by using overexpression of the UDP-glucose pyrophosphorylase 1 (Ugp1) that constituted the ubiquitin promoter, the outcome revealed astonishing findings that led to the development of the thermosensitive-genic-male sterility rice line due to Ugp1 silencing rather than overexpression. The silenced Ugp1 plants displayed normal pollen-mother-cells (PMC) before the meiosis stage, and later disruption of the callose deposition occurred during the meiosis. Consequently, the degeneration of the PMC at the meiosis beginning phase and leading to complete pollen development failure and plants showed male sterility phenotype. Therefore, Upg1 is signifying the role of sugar partitioning during the phase transition from sterility to fertility and stability under a fluctuating environment (Figure 5c). Recently, Chen et al. [41] reported that hms1 mutation caused abnormal lipid metabolism during male gamete development. The hms1 mutation significantly decreases very-long-chain-fatty-acids (VLCFAs) such as C26 and C28 and their derivates, which are integral constituents of the anther wax and pollen wall. Further, the examination of pollen walls of the hms1 and hms1i mutants displayed a reduction in bacula and tryphine layers. The HMS1 modulates the bacula and tryphine formation by interplaying with HMS1I to accelerate the C26 and C28 biosynthesis. The hms1 mutants [41] displayed male sterility and male fertility at low RH (<60%) and high RH (>80%), respectively (Figure 5b). These accumulating findings might emphasize understanding the novel regulatory mechanism of the male sterility in HR and can be utilized as references to explore and engineer more genes that are the key players during anther development.

#### 3.1.6. Transcription Factors Implicated in EGMS-Lines

Transcription factors (TFs) are proteins that regulate gene expression of many other genes via interacting or binding promoter regions or genes under environment signals. A functional understanding of TFs in EGMS systems or HR lines helps to a great extent to explore the complexity of adaptive controlling mechanisms of male fertility [28,151].

The transient expression of PTC1 in tapetal-cells and the microspores were reported. It regulates tapetal program cell death (PCD) and the production of pollen. Intriguingly, HengnongS-1 displays a stable switch of fertility in Indica (background rice), when it was grown under HT, pollen grain formation was disrupted and led to male sterility. However, the pollen grains were produced normally under normal temperature (25 °C-day and 23 °C-night). In contrast, PTC1′s second exon contains T insertion (single nucleotide) as well as lacks a PHD_motif in ptc1 mutants that revealed complete male sterility at low and high temperatures in Japonica cultivar-9522 [152]. A few DMR-linked genes, i.e., LOC_Os08g38210 (TFs BIM2) and LOC_Os06g40200 (chalcone-synthase) were influenced by DL in PA64S [153,154]. In another study, the photoperiod responsive gene OsPRR37 directly influences male sterility transition in the NK58S [154,155]. Recently, advances in HR technology as functional rice genomics and forward/reverse genetics laid a solid foundation to explore new innovative dimensions in recognizing and generating further valuable alleles for HR breeding. For instance, Zhang et al. [76] characterized the novel gene for the rPGMS, it displayed the carbon-starved-anther (CSA) phenotype. The assigned locus of the CSA was LOC_Os01g16810 that regulates the R2R3 TF and is specifically expressed among tapetum and vascular tissues. CSA regulates the transcript of the OsMST8 that is a transporter for monosaccharide [76]. Due to the mutation of CSA within R2R3 MYB-TFs reduced greatly transcript level of the OsMST8, disrupted sugar translocation from source (flag leaf to lemma or palea by the stem) to sink (anther), led to the trait of rPGMS, and showed fertility under LD conditions and complete male sterility under SD conditions (Figure 5a) in both Indica and Japonica (background rice) [75,76]. The CSA has shown great potential genetic resources to be applicable in creating new hybrids by two-line HR technology.

#### 3.1.7. Thermo-Sensitive-Male-Sterility Is Regulated by LRR-RLK

The leucine-rich-repeat receptor-like kinases (LRR-RLK) have been demonstrated to influence several events of plant growth and development and maintaining cell communication network by processing extracellular stimuli to the cytoplasm and/or nucleus. In the anther development, LRR-RLK play an important role in regulating defining movements of the tapetum-cells and meiocytes [156]. Few kinases including the RLKs as well as Hexokinase react as key regulators in tapetal-PCD. Yu et al. [43] reported that *TMS10* and its homolog as *TMS10L*, encode LRR-RLK and the tms10 mutant displayed male sterility to fertility transition at high to low temperature. The tms10 mutant displayed sterility phenotype due to expended and vacuolated tapetum at high-temperature, leading to the pollen grains abortion at the S9 stage. The tms10 or tms10L plants that contain a single mutation, showed male fertility at low-temperature, but the plants consisting of the *tms10* and *tms10L* double allelic mutations, were male sterile at high as well as low temperature, and such findings indicated functional redundancy. The transcript expression of *tms10L* was higher at low temperature, indicating that *TMS10L* plays an important role at low temperature. The present results suggest that both *TMS10* and *TMS10L* are monitoring switches for alterations of temperature in buffering anther development conditions at the late-stage of meiosis [43]. These attempts to elucidate the molecular mechanism of the EGMS systems may have imperative consequences for unraveling the molecular regulatory forces of the photoperiod, temperature, and humidity modulation of several biological processes, as well as the creation of the more male sterile germplasm by genetic manipulation for the enhancement of hybrid breeding.

#### 3.1.8. Development of EGMS Lines via CRISPR/Cas9 Technology

The genome editing techniques are vital tools in gene engineering for obtaining scientific objectives via introducing specific and precise DNA targets in vivo [157,158,159]. Zhou et al. [42] knocked out the *TMS5* gene to generate transgene clean TGMS-lines in one year by CRISPR/Cas9 technology. This move demonstrated that efficient editing technology embraces incredible future potential in reducing breeding period and efficiency. In rice, the *CSA* gene was edited with the same technology to produce two rPGMS-lines as JY5B and 9522csa1 to reveal male fertility to sterility and vice versa transition under changing photoperiod duration. Additionally, the *CSA* gene in Japonica background (Kongyu131, KY131) was edited to generate a KY131 mutant that was sensitive to both temperature and photoperiod, proposing allelic expression regulated differently in different genetic background germplasm for fertility and sterility transition in HR under environmental fluctuations [75,79]. Using CRISPR-Cas9 technology to edit the *TMS10* of other rice varieties also showed temperature-sensitive characteristics, which provides a genetic resource for cross-breeding [43]. The *p/tms12-1* locus showed dual traits in EGMS-lines (PGMS and TGMS), because of the disruption in the function of the osa-smR584m [49]. Ma et al. [160] revealed CRISPR/Cas9 technology with 85.4% average frequency of mutation in the rice genome and achieved 82% editing efficiency of the desire targets via insertion, deletion, inversion, and substitution [161,162]. Thus, it is a valuable editing system to generate modifications in genes to explore unrevealed mechanisms and produce sustainable genetic germplasm.

## 4. Apomixis Technology Could Be the Future of the Single-Line Hybrid Breeding

There is no uncertainty that HR contributes greatly to increase crop production, produced through the three-line and/or two-line systems, but this benefit of yield cannot be persistent in generation to generation due to genetic vulnerability. Therefore, the farmer communities need to invest or repurchase new seeds each year for better crop production. It is a very serious issue, that the prices of hybrid seed are increasing 10–15 times more as compared to the normal seeds, thus decreasing the probability margin of the farmers due to the use of expensive inputs [163]. Moreover, the farmers in the developing countries are unable to purchase expensive seeds and deprived of the benefits of hybrid breeding. The race for developing homozygous rice lines started in 1964 after the novel discovery of androgenic haploidy and successfully generated rice haploids through anther culture [164,165]. The anther culturing method was utilized to produce homozygous double-haploid (DH) rice lines and promoted the rice enhancement programs around the globe [166]. The recombinant DH generated using hybrid-rice displayed similar yield and improvement in grain quality as well as bypassed obstacles associated with HR technologies. Approximately 20 varieties of rice have been stated through DH method in China, India, Japan, Korea, and USA. There are also constraints such as embryogenic calli induction problem in genotypes, anther necrosis, low seedling generation, and common albino plant generation, etc., that hinder the application of the DH method at broad spectrum [167,168,169].

**Figure 6:**
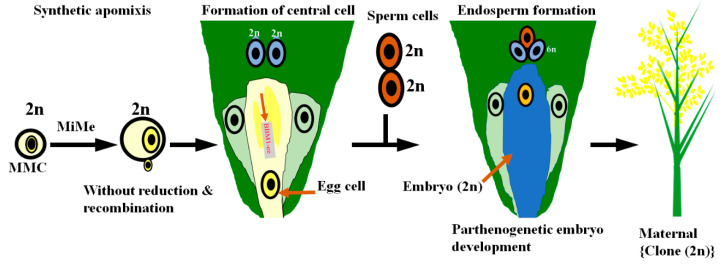
Apomixis technology for single-line HR.

Currently, more sustainability in seed production is a prerequisite that may be achieved by utilizing the recent advances in apomixes technology. Hybrids produced by apomixis can provide a bright opportunity to the farmers to multiply and cultivate future commercial crops on their own farm’s generation after generation. Apomixis will greatly improve hybrid breeding and can assist in achieving the breed’s dream to produce more germplasm that can be more suitable according to the cropping patterns or microenvironments and the true hybrids like pure breeds with less heritable vulnerability and [170]. This technology will greatly facilitate and reduce the time for seed production as well as save > USD 2.5 billion/annum cost of HR seed production. Then, the developing countries will also be able to gain benefits from the HR technology. Apomixis is a combination of the “apo” (away from) and “mixis” (act of mingling or mixing) and refers to the asexual mode of reproduction via seed lack of meiosis and fertilization [171]. In this reproduction method, the embryo (seed) advances in the absence of sperm and egg mating and this process circumvents female meiosis as well as syngamy to create embryos heritably alike to the maternal plant (Figure 6). As a result, during the utility of the apomixis, the F1 siblings will generate seeds that will be a true copy of the parent and secure the seed production [172,173,174].

Several examples indicate successful genetic manipulation via apomixis in fruits (mango, citrus, and mangosteen) and forage grasses (*Pennisetum*, *Brachiaria*, *Panicum*, and *Dichanthium*). Apomixis has been utilized in maize genetic manipulation through *Tripsacum dactyloides* germplasm (wild type maize parent) [170]. Therefore, practical implementation of the apomixis technology in crops can revolutionize the future of agriculture. For apomictic crops production, attempts were carried out in *Pennisetum squamulatum*, *Cenchrus ciliarisgene*, *Brassica napus*, and Arabidopsis by genetic manipulation of the *CcASGRBBML*, *PsASGRBBML*, *BnBBML*, and BBML genes, respectively [175,176]. Currently, rice genetic manipulation by BABY BOOM engineering induced parthenogenesis, e.g., BBM1-ee (Figure 6).

MiMe produce an egg (2n) that is unrecombined and unreduced due to lack of meiosis. This egg (2n) cell is parthenogenetically transformed into embryo (clone) via BBM1-ee and results in the fertile plant that is a maternal copy (single-line HR).

The fractional apomixis has also been accomplished in rice through the OsBBM gene [177,178]. Functional characterization of the EGMS genes and utilization during the apomixis technology will be a plausible potential approach to generate the single-line hybrids in cereal crops.

## 5. Conclusions

The MS systems are the essence to explore the MF mechanism for creating the two-line HR. Two-line HR is becoming the hot topic of research due to several advantages over the three-line system in utilization for hybrid breeding all over the world. The sterility to fertility transition, and vice versa, regulated by the EGMS systems (PGMS, TGMS, and HGMS) were regulated via environmental fluctuations (photoperiod and/or temperature and humidity) in HR. Yet, a limited number of EGMS genes are cloned and their mechanism is regulated by modifications at the RNA-metabolism and structural substances. The characterization of the EGMS genes/systems and harnessing apomixis technology for seed or single-line hybrids production will emphasize agriculture dynamic shifts.

## 6. Future Perspectives of EGMS Research and Application

Agriculture is intensifying due to the increasing world population. More stable and consistent development in technology can ensure food supply and attain food security, especially in developing countries. Research development in rice functional genomic allowed scientists to characterize MS responsible factors/genes in the current era and create rice EGMS-lines. The outcomes highlighted that the functional nature of EGMS genes is greatly variable in the comparative aspect. Herein, we revealed in detail MS systems used for generating rice EGMS-lines and elaborated potential gaps that could be minimized by creating more genetic resources by functional characterization of target genes in the near future. Target specific gene engineering according to the required condition for producing more EGMS resources will shed more light on existing unrevealing regulatory mechanisms during male sterility in plants. Currently, there is no doubt that we have a lot of EGMS resources in two-line HR under cultivation, but few genes responsive to the photoperiod and/or temperature have been cloned. Yet, the major problems need more consideration, such as male sterility instability and inadequate restoration of pollen fertility (male fertility). The male sterility instability occurs due to abrupt fluctuations in the environment (photoperiod, temperature, and humidity) under field conditions, facilitates self-crossing that results in unwanted seed setting, and increase seed impurity that trigger decline in HR yield. The male fertility restoration problem occurs under desirable conditions for the multiplication of the male sterile (EGMS-lines) resources.

Previously, it was disclosed that approximately 20 photo-thermosensitive-genic-male sterile genes were described among different rice genetic resources. To date, *pms1, pms3,* or *p/tms2-1* and *CSA* and *tms5*, *tms10*, and *ugp1* genes responsive to the photo-sensitive-genic-male sterility and thermosensitive-genic-male sterility, respectively, have been cloned and investigated for unrevealing regulatory mechanisms in two-line HR. Additionally, literature and genomic resource availability of the functionally characterized TGMS genes from *Arabidopsis thaliana* may help in mining more TGMS genes for application in the development of two-line HR. In future research, functional characterization will be carried out for more genes, to extend knowledge of understanding about EGMS lines. The photo-sensitive-genic-male-sterile genes also are vulnerable to genetic drift and temperature fluctuations. In contrast to temperature, theoretically, photoperiod seems more stable and seed production can be a safe option by using photo-sensitive-genic-male sterile lines. However, light-responsive genes are often susceptible to the effect of temperature and other genes, as a result, the photo-sensitive sterile genes are also unstable. As the photoperiod responsive sterile gene derived by NK58 by using Japonica rice is prone to genetic drift and does not produce stable offspring, but it shows the TGMS trait when transferred to PA64S, Indica rice. Similarly, CSA displays photoperiod response in 9522 (Japonica background), it displays P/TGMS trait, the dual response in Kongyu131 (Japonica background) against photoperiod and temperature. Therefore, more understanding of the regulatory molecular mechanism needs to be elucidated. At last, it is an open and remaining challenge on how to obtain stable and well-defined critical sterility inducing condition (CSIC) for the development of EGMS lines to ensure the safety of seed production in hybrids. Many varieties of TGMS lines (Japonica background) have high critical sterility inducing temperature, which mainly restricts the application of two-line HR. So far, the characterization of more EGMS genes for finding CSIC and determining molecular mechanisms have a great influence for the two-line HR application. Functionally characterized genes in two-line HR can be a practically useful material to optimize apomixis technology and explore the molecular mechanism. Besides, present elevated improvement in research of rice functional genomic as well as gene engineering will be an important milestone in achieving future goals, to develop HR germplasm, and gain eco-friendly sustainable agriculture.

## Figures and Tables

**Table 1:** Characterization of fertility restoring *Rf* genes.

Sr. #	Rf Locus in CMS Line for Three-Line HR Technology	References
1	*Rf4* identified in CMS-wild-abortive (WA) and classified as PPR protein	[30,32]
2	*Rf1a*, *Rf1b* (*Rf5*) identified in CMS-Chinsurah-Boro II/Taichung 65 (BT) and classified as PPR protein	[33,34]
3	*Rf2* identified in CMS Lead-rice (LD) and classified as non-PPR protein with glycine-rich domain	[29]
4	*Rf-A619* region identified in CMS-Charrua (CMS-C) with unknown protein character	[35]
5	*Rf5* (*Rf1b*), Rf6 identified in CMS-Honglian (HL) and classified as PPR protein	[30,31]
6	*Rf17* identified in CMS Chinese wild-type rice (CW) and classified as Acyl-synthase a carrier protein	[36]

**Table 2:** The prominent characters of the key EGMS lines and regulating factor/gene.

Sr. #	EGMS Line	Locus/Genes Responsive for EGMS Lines in Rice	References
1	NK58S	*PMS1, PMS2, PMS3* generate PGMS ^1^ in Japonica	[51,65,66]
2	Mian9S	*PMS4* generates PGMS ^1^ in Indica	[77]
3	Yi D1S	*RPMS1* and *RPMS2* generate rPGMS ^2^ in Indica	[73,78]
4	9522csa	*CSA* generates rPGMS ^2^ in Japonica	[79]
5	5460S	*TMS1* generates TGMS ^3^ in Indica	[80,81]
6	AnnongS-1	*TMS5* generates TGMS ^3^ in Indica	[46]
7	HengnongS-1	*TMS9-1* generates TGMS ^3^ in Indica	[82]
8	Zhu1S	*TMS9* generates TGMS ^3^ in Indica	[83,84,85]
9	NorinPL12	*TMS2* generates TGMS ^3^ in Japonica	[86]
10	IR32364	*TMS3(t)* generates TGMS ^3^ in Indica	[87]
11	TGMS-VN1	*TMS4(t)* generates TGMS ^3^ in Indica	[88]
12	Sokcho-MS	*TMS6* generates TGMS ^3^ in Japonica	[89]
13	SA2	*TGMS* generates TGMS ^3^ in Indica	[90]
14	J207S	*RTMS1* generates TGMS ^3^ in Indica	[91]
15	G20S	*TMS6(t)* generates TGMS ^3^ in Japonica	[92]

Abbreviations: ^1^ photoperiod-sensitive-genic-male-sterility; ^2^ reverse-photoperiod-sensitive-genic-male-sterility; ^3^ temperature-sensitive-genic-male-sterility.

**Table 3:** Regulation checkpoints of the EGMS systems for fertility transition in EGMS lines.

Sr. #	Regulation Point of EGMS System in EGMS Line	References
1	PGMS ^1^, DL ≤ 13 h (MF), ≥ 13.75 h (MS) in NK58S	[68,69,74]
2	TGMS ^2^, LT ≤ 23.5 °C (MF), HT ≥ 27 °C (MS) in PA64S	[78,119]
3	rPGMS ^3^, HT ≥ 13.5 h (MF), LT ≤ 12.5 (MS) in CSA	[75]
4	TGMS ^2^, LT ≤ 21 °C (MF), HT ≥ 28 °C (MS) in Ugp1	[57]
5	TGMS, LT ≤ 23.5 °C (MF), HT ≥ 27 °C (MS) in 93-11s	[42]
6	HGMS ^4^, RH > 80% (MF), RH < 60% (MS) in E157 and S4928	[54]
7	HGMS ^4^, RH > 80%(MF), RH 30–60% (MS) in osgl1-4	[120]
8	HGMS ^4^, RH > 75%(MF), RH = 45% (MS) in hms1	[41]
9	TGMS ^2^, TGMS = 22–24 °C (MF), >24 °C (MS) in tms10	[43]
10	PTGMS ^5^, LD (14 h) and SD (12 h) conditions or HT (27–30 °C) and LT (21–23 °C) in p/tms12-1	[42]
11	PGMS ^1^ ≤ 13 h (MF), ≥13.75 h (MS) in YiD1S	[73]

Abbreviations: ^1^ photoperiod-sensitive-genic-male-sterility, ^2^ temperature-sensitive-genic-male-sterility, ^3^ reverse-photoperiod-sensitive-genic-male-sterility, ^4^ humidity-sensitive-genic-male-sterility, ^5^ photo-thermo-sensitive-genic-male-sterility. The letters MF and MS in parentheses indicate male fertility and male sterility as well as, the DL, LH, and HT represent day length, low temperature, and high temperature, respectively.
